# The added value of CA125 normalization before interval debulking surgery to the chemotherapy response score for the prognostication of ovarian cancer patients receiving neoadjuvant chemotherapy for advanced disease

**DOI:** 10.7150/jca.52711

**Published:** 2021-01-01

**Authors:** Wei-feng Liang, Li-juan Wang, Hui Li, Chang-hao Liu, Miao-fang Wu, Jing Li

**Affiliations:** 1 Department of Gynecology and Obstetrics, Qilu Hospital (Qingdao), Cheeloo College of Medicine, Shandong University, Qingdao, 266035, People's Republic of China.; 2 Department of Gynecologic Oncology, Sun Yat-sen Memorial Hospital, Sun Yat-sen University, Guangzhou, 510120, People's Republic of China.

**Keywords:** biomarkers, chemotherapy, gynecologic oncology, prognosis

## Abstract

**Objective:** To investigate whether CA125 normalization following neoadjuvant chemotherapy (NACT) can complement the chemotherapy response system (CRS) in the prognostication of patients with tubo-ovarian high-grade serous carcinoma (HGSC).

**Methods:** In total, 118 HGSC patients who received NACT followed by interval debulking surgery (IDS) for FIGO stage IIIC-IV disease were included, and their clinical data were retrospectively reviewed. The primary endpoint was progression-free survival (PFS). Cox regression analysis was performed to identify predictors of PFS.

**Results:** Following NACT, CRS3 was noted in 35 patients (29.7%), and CA125 normalization (≤ 35 U/ml) was noted in 54 patients (45.8%). Both CRS3 and CA125 normalization were identified as independent prognosticators of PFS. Combining these two factors, we stratified the 106 patients into three groups with different risks of recurrence: low-risk group (CRS3 + post-NACT CA125≤ 35 U/ml; n = 17, 14.4%), intermediate-risk group (CRS3 + post-NACT CA125 > 35 U/ml; n = 19, 16.1%) and high-risk group (CRS1-2; n= 82, 69.5%). The differences in PFS between the three groups were significant (log-rank test, *P* < 0.0001). In Cox regression analyses, the new stratification method was found to have an independent prognostic effect.

**Conclusion:** Both the CRS system and the normalization of CA125 following NACT could reliably predict the risk of recurrence following primary treatment. The combination of the two factors refined the prognostic stratification of HGSC patients who were treated with NACT and IDS.

## Introduction

Ovarian cancer is the most lethal gynecological cancer with 52,100 new cases and 22,500 deaths estimated in China in 2015 1. Primary debulking surgery (PDS) followed by platinum-based chemotherapy is the standard treatment for ovarian cancer 2. However, even among patients who have no evidence of disease following chemotherapy, 70% experience relapse within the subsequent three years 3. Cytoreduction to no gross residual disease (R0 resection) can significantly improve the prognosis of ovarian cancer patients. Therefore, it is currently well established that the ultimate goal of PDS is R0 resection, and maximal surgical effort should be made to this 2. Aggressive debulking surgery is morbid, with a 20-25% major complication rate and a 1-2% mortality rate 4-6. Given this information, for patients with nonresectable disease or those who are considered unable to tolerate PDS, neoadjuvant chemotherapy (NACT) with interval debulking surgery (IDS) is an acceptable alternative 2,3. Supporting evidence for this treatment comes from three randomized controlled trials (RCTs) that compared NACT-IDS with PDS and reported that the prognosis of patients treated with NACT-IDS was noninferior to that of patients treated with PDS 7-9. However, these trials have been criticized because the progression-free survival (PFS), OS and optimal debulking rates were lower than those reported in previous studies 10. Therefore, the use of NACT remains under debate.

In the 2019 European Society for Medical Oncology (ESMO) ovarian cancer guidelines, a three-tiered chemotherapy response score (CRS) system was recommended for patients receiving NACT to evaluate tumor response and predict prognosis 11. Since its description, the CRS system has been independently assessed in many studies 12-17. Currently, it is considered an accurate and highly reproducible method to predict survival outcomes for patients with tubo-ovarian high-grade serous carcinoma (HGSC) 11. Recently, the HGSC CRS Collaborative Network validated the prognostic role of the CRS system in a real-world, heterogeneous study population 15. Pooling individual patient data from 11 countries, the authors reported that CRS3 was independently associated with an improved prognosis. Based on the findings, the CRS system is proposed as a surrogate for both PFS and OS 15.

For HGSC patients who are treated with NACT-IDS, another important prognostic marker is CA125. Considering that the early normalization of CA125 is associated with improved oncological outcomes 18,19, we hypothesized that combining CA125 normalization and CRS would further refine the prognostic stratification of HGSC patients and identify patients who could gain the most from the NACT-IDS treatment modality.

## Patients and Methods

This was a retrospective cohort study. The primary objective was to investigate whether the normalization of CA125 (≤ 35 U/ml) before IDS could add prognostic value to the CRS system to predict PFS in HGSC patients treated with NACT-IDS. The data were collected from two tertiary-referral university hospitals in China. The study protocol was approved by the Institutional Review Board (approval NO. SYSEC-KY-KS-2020-088). Patient charts were reviewed to identify those with International Federation of Gynecology and Obstetrics (FIGO) stages IIIC-IV ovarian, fallopian tube, or primary peritoneal HGSC who received NACT-IDS at either hospital during the period from January 2012 to November 2019.

The decision to recommend PDS was made based on the likelihood of achieving R0 resection and patient tolerance to the surgery. A multidisciplinary team (MDT), including two experienced gynecological oncologists, one pathologist and one radiologist, assessed the possibility of R0 resection. Pre-NACT biopsy was obtained from all patients and reviewed by two pathologists to confirm the histological type. Patients who did not complete the primary treatment or had received chemotherapy at an outside institution were excluded from analysis. NACT regimens were largely platinum- and paclitaxel-based and reflected the standard protocols in practice guidelines during the study period. According to the Society of Gynecologic Oncology (SGO) and American Society of Clinical Oncology (ASCO) guidelines 20, patients with a response to NACT or stable disease underwent IDS after ≤ 3 cycles of NACT. IDS was performed via a midline laparotomy by experienced gynecologic oncologists. For patients considered to have tumor progression during NACT, IDS was not considered; they were treated with second-line chemotherapy regimens and were excluded from the final analysis. All patients received a minimum of six cycles of chemotherapy, which included at least three cycles of adjuvant chemotherapy following IDS 2,20. Serum levels of CA125 were routinely measured at diagnosis, before each cycle of chemotherapy and at IDS. Written informed consent was obtained from all patients.

The pathology slides of specimens from IDS were independently reviewed by two pathologists. CRS analysis was performed based on omental samples, which was in line with the International Collaboration on Cancer Reporting (ICCR) guidelines 21. In the case of disputes concerning CRS classification, a unanimous agreement was reached after sample re-evaluation.

Patients were seen in routine follow-up every three months for two years after primary treatment, every six months for the following three years, and every year thereafter. Follow-up visits included a gynecologic examination and measurements of tumor markers. Surveillance ultrasonography examination and computed tomography (CT) and magnetic resonance imaging (MRI) scans were performed at the discretion of the gynecologic oncologist. PFS was defined as the interval from the date of the completion of primary treatment to the date of recurrence. OS was defined as the interval from the date of the completion of primary treatment to death.

We calculated descriptive statistics including medians and portions. PFS and OS were evaluated using the Kaplan-Meier method and compared with the log-rank test. Cox proportional hazard models (enter method) were fitted to estimate hazard ratios (HRs) and 95% confidence intervals (95% CIs) for PFS and OS. For multiple comparisons of survival curves, the Bonferroni adjustment was applied. All statistical tests were two-sided, and differences were considered significant at *P* < 0.05. STATA 12.0 (Stata Press, College Station, TX, USA) and MedCalc 17.0 (MedCalc Software Ltd, Ostend, Belgium) were used for all analyses.

## Results

### Patient characteristics

A total of 118 patients were included in the final analysis. **Table 1** shows the patient demographics and treatment characteristics. All 118 patients received three cycles of NACT. The median time interval between the last cycle of NACT and IDS was 25 days (range: 22 to 28 days). Before NACT, all patients were noted to have elevated levels of CA125 (median: 1218.9 U/ml, range: 106.4 to 17354.0 U/ml). Following NACT, CA125 ≤ 35 U/ml was documented in 54 patients (45.8%). Following NACT, CRS3 was noted in 35 patients (29.7%). Of the 35 CRS3 patients, CA125 normalization was noted in 17 (48.6%) patients. Supplementary Table 1 details CA125 status following NACT among CRS3 patients based on the presence or absence of complete pathological response (pCR). CA125 normalization was more frequently observed in CRS3 patients with pCR than CRS3 patients with near-complete response or minimally residual tumor (72.7% vs. 37.5%); the difference was marginally significant (*P* = 0.053). All CRS3 patients underwent R0 cytoreduction in IDS and received three cycles of adjuvant chemotherapy after IDS. Of the 83 patients who did not achieve CRS3, 64 (77.1%) underwent R0 debulking surgery, and 71 (85.5%) received three cycles of chemotherapy following IDS.

### Survival outcomes stratified by CRS and CA125 normalization

**Figure 1:**
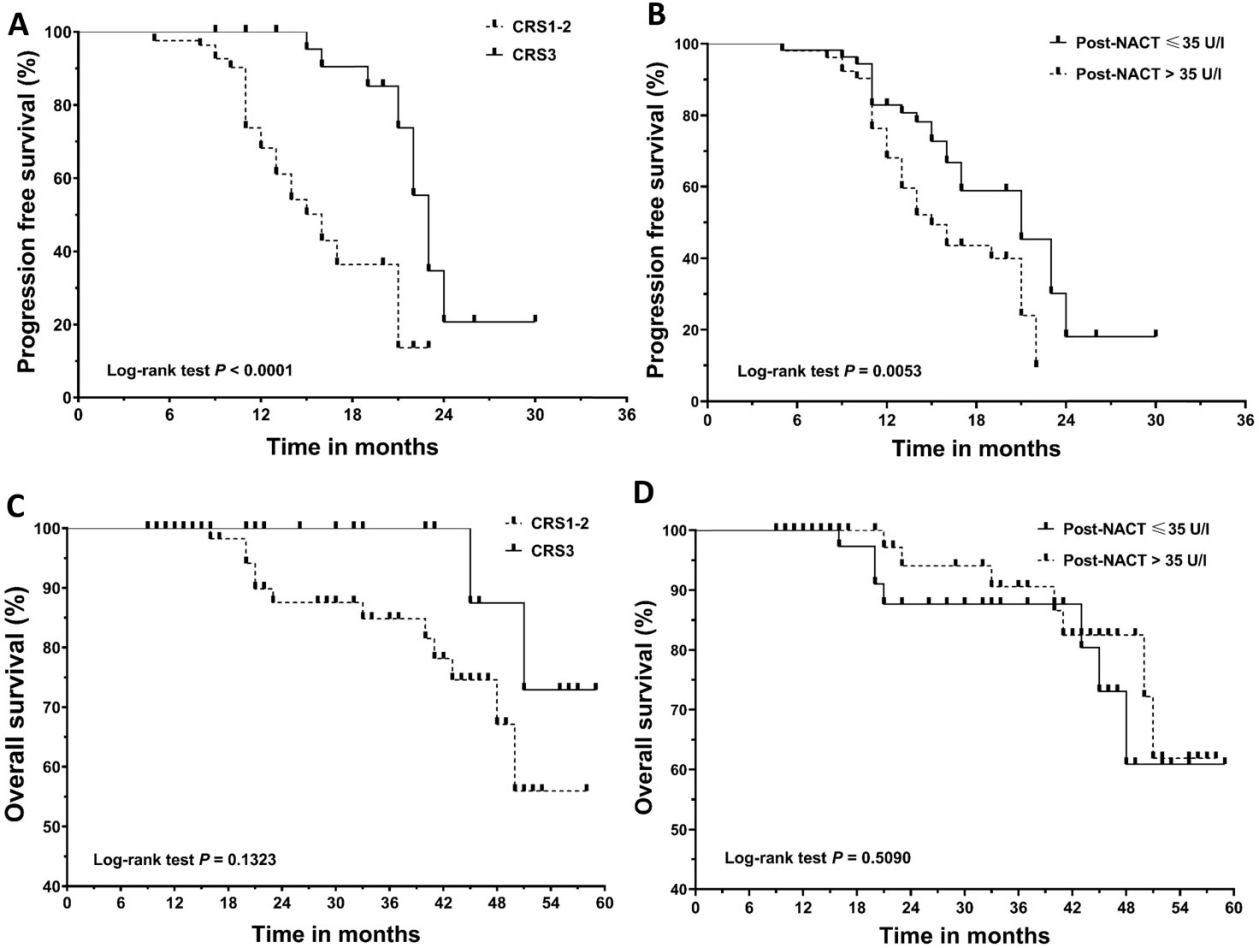
Kaplan‐Meier curves for progression‐free survival (PFS) and overall survival (OS). (A) PFS according to chemotherapy response score (CRS). (B) PFS according to post-NACT CA125 level. (C) OS according to chemotherapy response score (CRS). (D) OS according to post-NACT CA125 level. NACT, neoadjuvant chemotherapy.

The median follow-up for the cohort was 21.0 months (range: 9 to 59 months). Recurrence and death were documented in 66 patients and 14 patients, respectively. **Figure 1** shows the survival curves for PFS and OS. CRS3 (HR=0.34; 95% CI, 0.19 to 0.62; median PFS, 22 vs 16 months) and normalization of CA125 following NACT (HR=0.51; 95% CI, 0.30 to 0.88; median PFS, 21 vs 16 months) were associated with a decreased risk of recurrence. Median OS was not achieved in our cohort. The difference in OS between the groups categorized by CRS3 (HR = 0.32; 95% CI, 0.07 to 1.45; median OS not achieved) and post-NACT CA125 (HR = 1.45; 95% CI, 0.50 to 4.15; median OS not achieved) did not reach significance. **Table 2** summarizes the results of the univariate and multivariate Cox analyses. CRS3, normalization of CA125 following NACT and R0 resection were identified as independent prognosticators for PFS. Considering the importance of CA125 normalization, we conducted a subgroup analysis in patients who achieved CRS3 following NACT and the results are summarized in **Table 3.** After adjusting for other covariants, CA125 normalization was independently associated with a decreased risk of recurrence (HR = 0.08; 95% CI, 0.02 to 0.45; *P* = 0.004).

### The added prognostic value of normalization of CA125 to CRS system

**Figure 2:**
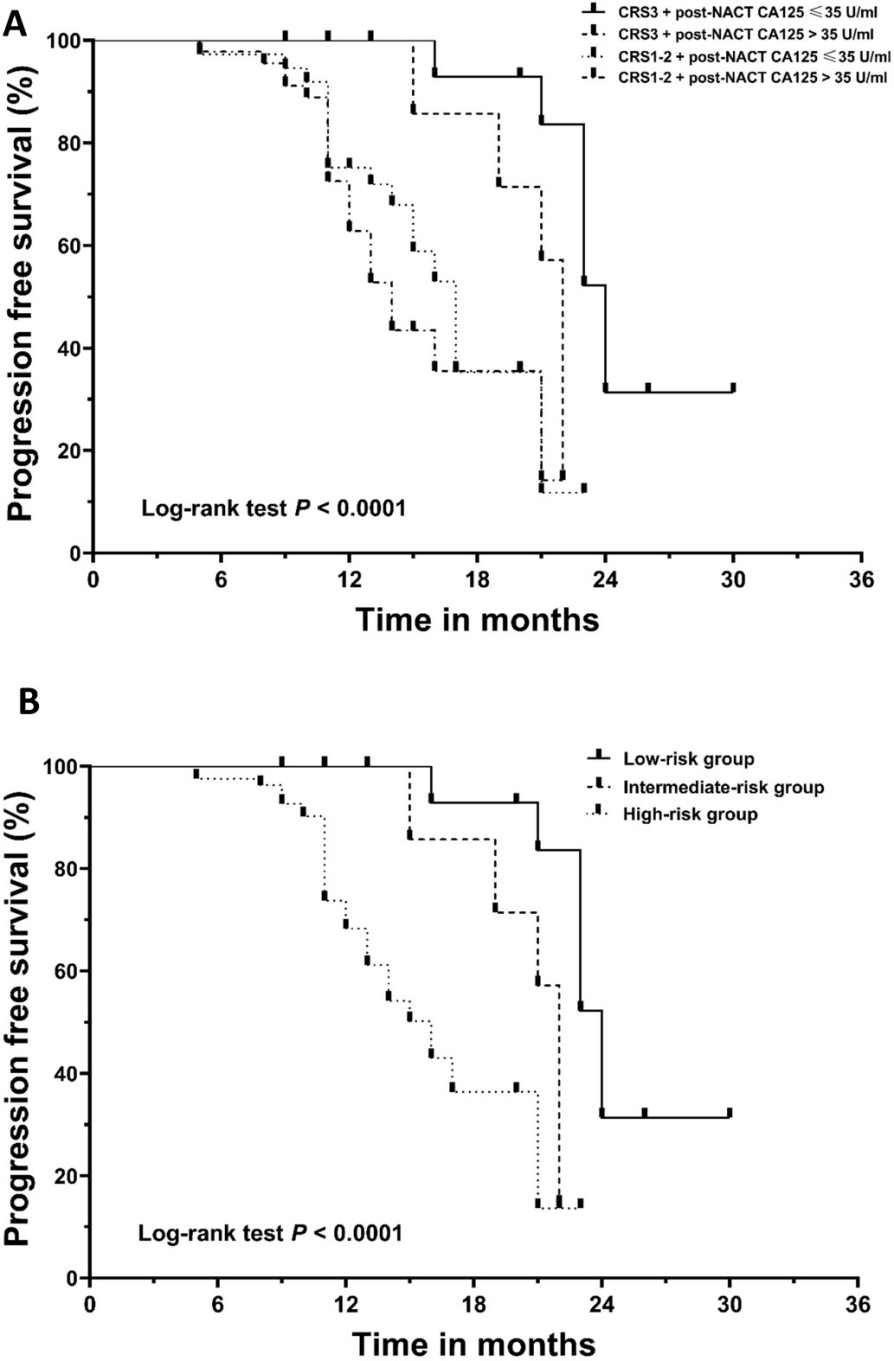
Kaplan‐Meier curves for progression‐free survival (PFS). (A) The patients were divided into four subgroups based on their chemotherapy response score (CRS) and post-NACT CA125 level. (B) The patients were stratified into low-, intermediate-, and high-risk categories. NACT, neoadjuvant chemotherapy.

Given the independent prognostic importance of CRS3 and the normalization of CA125 for PFS, we conducted further analysis to assess a new classification system by integrating the two factors. The cohort was divided into four subgroups: CRS3 + post-NACT CA125≤ 35 U/ml (n = 17, 14.4%), CRS3 + post-NACT CA125 > 35 U/ml (n = 18, 15.3%), CRS1-2 + post-NACT CA125 ≤ 35 U/ml (n = 37, 31.4%) and CRS1-2 + post-NACT CA125 > 35 U/ml (n = 46, 39.0%). **Figure 2A** demonstrates the survival curve for PFS; the differences between the four groups were significant (log-rank test, *P* < 0.0001). Supplementary Table 2 summarizes the results of the post hoc Bonferroni analysis. Since the difference between the group with CRS1-2 + post-NACT CA125 ≤ 35 U/ml and the group with CRS1-2 + post-NACT CA125 > 35 U/ml did not reach significance, we combined the two groups. Finally, using a combination of the CRS system and post-NACT CA125, we classified the 118 patients into three groups: low-risk group (CRS3 + post-NACT CA125≤ 35 U/ml; n = 17, 14.4%), intermediate-risk group (CRS3 + post-NACT CA125 > 35 U/ml; n = 19, 16.1%) and high-risk group (CRS1-2; n= 82, 69.5%). Kaplan-Meier curves for PFS are shown in **Figure 2B**; the differences between the three groups were significant (log-rank test, *P* < 0.0001). **Table 4** summarizes the results of the post hoc Bonferroni analysis. The differences between the low-risk group and the intermediate-risk group (log-rank test, *P* = 0.001), the low-risk group and the high-risk group (log-rank test, *P* < 0.0001), and the intermediate-risk group and the high-risk group (log-rank test, *P* = 0.040) were statistically significant. A Cox analysis was conducted where the low-risk group was used as a reference, and the results are shown in **Table 5.** After adjusting for other prognosticators, the HRs for PFS of the intermediate-risk group and high-risk group were 4.54 (95% CI, 1.47 to 14.00; *P* = 0.008) and 6.10 (95% CI, 2.14 to 17.40; *P* = 0.001), respectively.

## Discussion

Whether NACT-IDS is noninferior to PDS regarding patient prognosis has been a controversial topic. Approximately 82% of SGO members did not consider that there is sufficient evidence to justify NACT 22. Despite this, the utility of NACT in the United States increased from 7.7% in 2004 to 27.8% in 2015 23. NACT can decrease tumor volume, thereby allowing for a less traumatic surgery and a higher R0 resection rate 20. However, this treatment exposes a high tumor burden to chemotherapeutic drugs, which results in a selection of resistant tumor clones 24,25. Even among HGSC patients with BRCA1-heterozygous tumors that are supersensitive to DNA-damaging drugs and poly (ADP-ribose) polymerase (PARP) inhibitors, the utility of NACT is observed to facilitate the expansion of pre-existing BRCA1-proficient tumor clones 26. In addition, there have been clinical data suggesting that NACT patients are more likely to develop platinum-resistant recurrence 27,28. The Japan Clinical Oncology Group phase III RCT 0602 (JCOG0602) compared NACT-IDS with PDS 29. In 2020, the authors reported that the survival noninferiority of NACT was not validated in the trial 29. At the Annual Meeting of ASCO in 2020, using data from the PAOLA-1 trial, C. Grimm et al. reported that among ovarian cancer patients receiving olaparib plus bevacizumab as maintenance therapy, the magnitude of PFS benefit was lower in NACT patients treated with NACT than in patients treated with PDS 30. Collectively, these findings remind us of the potential negative influence of NACT. We believe that patients treated with NACT should be stratified according to their prognosis so they can have a better opportunity to gain a survival benefit from more individualized management.

The CRS system can divide HGSC patients who receive NACT according to their response: complete/near complete (CRS3), partial (CRS2), and no/minimal (CRS1) responses. Since the survival outcomes of the CRS1 patients were similar to those of the CRS2 patients, CRS can actually be treated as a binary prognostic system that stratifies patients into two subgroups 12,17. Herein, we observed that CRS3 could be achieved in 29.7% of NACT patients, and this cohort had a significantly decreased risk of recurrence compared with those who achieved CRS1-2. These results are consistent with those of previous studies and confirmed that the CRS system can be used as a reliable tool for prognostic stratification 15,17. In the study by the HGSC CRS Collaborative Network, more patients in the CRS3 group were noted to have a germline *BRCA* 1/2 mutation than those in the CRS1-2 group 15. Therefore, CRS3 following NACT may suggest a favorable tumor biology, which provides a possible explanation for why CRS3 patients benefit more from NACT than the others.

In the neoadjuvant setting, CA125 can be utilized as a marker to assess the response to chemotherapy. The post-NACT CA125 level can predict the possibility of achieving optimal cytoreduction in IDS 31-33. Despite this, the CA125 response does not exactly equate to the pathological response 12,15. As we observed, CA125 normalization was noted in 48.6% of patients achieving CRS3. We identified the normalization of CA125 following NACT as an independent predictor of decreased recurrence risk. Even in the CRS3 subgroup, patients with post-NACT CA125 ≤ 35 U/ml were observed to have a longer median PFS than those with post-NACT > 35 U/ml, which was further confirmed in the Cox analysis. These findings were not completely consistent with those in previous studies 31,34-37. However, given the following limitations in previous studies, caution must be taken in interpreting their results. First, many of the published studies included patients with non-serous epithelial cancer patients, yet the prognostic value of CA125 for these patients remains controversial 38. Second, there is convincing evidence that NACT will exert a negative effect on patient prognosis if the number of NACT cycles exceeds four 37,39. Although previous studies included patients receiving more than four cycles of NACT, the researchers did not consider or adjust the impact of the number of chemotherapy cycles. In light of these limitations, we believe that our findings complement those of prior studies and provide more reliable information regarding the prognostic role of CA125 normalization in NACT patients.

Since both the CRS system and the normalization of CA125 were independently associated with patient prognosis, we combined them and developed a new stratification method. Compared with the CRS system 12, the new method identified one more subgroup, the intermediate-risk group. Patients in the intermediate-risk group achieved CRS3 and received the same subsequent treatment as the other patients who achieved CRS3; however, they had a high risk of recurrence. The CRS system is based on omental assessment. Of note, the response of the omentum to NACT is not necessarily in line with that of other sites 12,15. Therefore, CRS3 patients are a heterogeneous group with varying tumor loads. Since the CA125 level is correlated with tumor burden in 93% of ovarian cancer patients 40, it could help identify the cohort of patients with a higher tumor load among the CRS3 patients, thereby complementing the CRS system.

The present study is the first one to explore the role of combining the CRS system and the CA125 level in an attempt to refine the prognostic stratification of HGSC patients who were treated with NACT. Given that the CRS and CA125 are readily available in clinical practice, we believe that our findings are easily applicable in the care of patients in resource-limited areas. Nevertheless, some limitations of the current study should be acknowledged. First, as a retrospective study, missing data could not be avoided, so the adjustment variables entered in the multivariable analyses might be incomplete. Second, since most health insurance plans in China do not cover the costs of genetic testing, information about *BRCA* 1/2 mutations was not available in most of the included patients, and we could not conduct a more detailed subgroup analysis. Third, although the PFS of our cohort was in line with that of previous reports 12,14, the follow-up period in the present study was relatively short, and the median OS was not reached. Finally, the sample size of this work is limited. The data of the present study were collected from two tertiary-referral university hospitals in China. The clinical decisions in our institutions are made in accordance with the ASCO-SGO guidelines 20, and NACT has been prescribed to only carefully selected patients. Thus, although we pooled eight years of data, only 118 patients were included in the final analysis.

In conclusion, this study confirmed that the CRS system has prognostic significance. In addition, we found that CA125 normalization following NACT is an independent prognosticator, and it can provide complementary information to the CRS system for predicting the prognosis of HGSC patients who are treated with NACT. The combined use of the two methods could help physicians better stratify patients and make individualized therapeutic decisions. More studies are needed to validate these findings.

## Supplementary Material

Supplementary tables.

## Figures and Tables

**Table 1:** Baseline characteristics

Variable	
Age (years), median (range)	58 (37 to 78)
BMI (kg/m^2^), median (range)	22.3 (19.0 to 27.1)
**FIGO stage, n (%)**	
IIIC	104 (88.1)
IV	14 (11.9)
**ECOG performance status, n (%)**	
Normal activity	104 (88.1)
Restricted activity	14 (11.9)
**BRCA status including germline and somatic, n (%)**	
BRCA1 mutant	3 (2.5)
BRCA2 mutant	5 (4.2)
BRCA wide type	8 (6.8)
Data unavailable	102 (86.4)
**R0 resection, n (%)**	
Yes	99 (83.9)
No	19 (16.1)
**ICU stay following IDS (%)**	
Yes	5 (4.2)
No	113 (95.8)
**NACT regimen (%)**	
Three-week carboplatin/paclitaxel	113 (95.8)
Weekly carboplatin/paclitaxel	5 (4.2)
**Chemotherapy response score**	
1	55 (46.6)
2	28 (23.7)
3	35 (29.7)
**CA125 (U/ml), median (range)**	
Pre-NACT	1218.9 (106.4 to 17354.0)
Post-NACT	38.4 (7.4 to 2292.0)
Normalization following NACT (≤ 35 U/ml), n (%)	54 (45.8)
**HE4 (pmol/l), median (range)**	
Pre-NACT	616 (105 to 6397)
Post-NACT	105 (19 to 1879)
**HGB (g/l), median (range)**	
Pre-NACT	98 (69 to 121)
Post-NACT	106 (85 to 125)
**Albumin (g/l), median (range)**	
Pre-NACT	24 (16 to 37)
Post-NACT	33 (26 to 41)

BMI, body mass index; CRS, chemotherapy response score; ECOG, eastern cooperative oncology group; FIGO, The International Federation of Gynecology and Obstetrics; HE4, Human epididymis protein 4; HGB, hemoglobin; ICU, intensive care unit; IDS, interval debulking surgery; NACT, neoadjuvant chemotherapy.

**Table 2:** Univariate and multivariate progression-free survival analyses

	Univariate analysis			Multivariate analysis		
	HR	95% CI	*P* value	HR	95% CI	*P* value
Age (Year)	1.01	0.98 to 1.05	0.433			
Stage (FIGO IIIC vs IV)	0.92	0.46 to 1.81	0.800			
BMI (kg/m^2^)	1.04	0.91 to 1.19	0.587			
ECOG (Normal activity vs Restricted activity)	1.30	0.68 to 2.48	0.430			
CRS3 (Yes vs No)	0.34	0.19 to 0.62	<0.0001	0.47	0.25 to 0.89	0.020
Post-NACT CA125<35 U/ml (Yes vs No)	0.51	0.30 to 0.88	0.015	0.49	0.28 to 0.84	0.010
Post-NACT HE4 (pmol/l)	1.00	0.9998 to 1.002	0.105			
Post-NACT albumin (g/l)	1.04	0.97 to 1.12	0.231			
Post-NACT HGB (g/l)	0.96	0.94 to 0.99	0.011	0.0.99	0.96 to 1.02	0.663
ICU (Yes vs No)	0.54	0.17 to 1.79	0.316			
R0 resection in IDS (Yes vs No)	0.03	0.01 to 0.09	< 0.0001	0.04	0.01 to 0.12	< 0.0001

BMI, body mass index; CI, confidence interval; CRS, chemotherapy response score; ECOG, eastern cooperative oncology group; FIGO, The International Federation of Gynecology and Obstetrics; HE4, Human epididymis protein 4; HGB, hemoglobin; HR, hazard ratio; ICU, intensive care unit; NACT, neoadjuvant chemotherapy.

**Table 3:** Multivariate progression-free survival analysis of patients achieving R0 resection in interval debulking surgery

	HR	95% CI	*P* value
Age (Year)	1.08	0.98 to 1.20	0.127
Stage (FIGO IIIC vs IV)	0.61	0.17 to 2.11	0.432
BMI (kg/m^2^)	1.41	0.92 to 2.17	0.115
ECOG (Normal activity vs Restricted activity)	0.47	0.04 to 5.12	0.539
Post-NACT HE4 (pmol/l)	1.00	0.99 to 1.01	0.625
Post-NACT albumin (g/l)	1.13	0.93 to 1.37	0.214
Post-NACT HGB (g/l)	1.01	0.94 to 1.09	0.738
Post-NACT CA125 (U/ml)	0.08	0.02 to 0.45	0.004
ICU (Yes vs No)	0.56	0.11 to 2.84	0.488

BMI, body mass index; CI, confidence interval; CRS, chemotherapy response score; ECOG, eastern cooperative oncology group; FIGO, The International Federation of Gynecology and Obstetrics; HE4, Human epididymis protein 4; HGB, hemoglobin; HR, hazard ratio; ICU, intensive care unit; NACT, neoadjuvant chemotherapy.

**Table 4:** Comparison of recurrence-free survival using the log-rank test with Bonferroni correction

	Low-risk group		Intermediate-risk group		High-risk group	
	Chi-Square	*P* value	Chi-Square	*P* value	Chi-Square	*P* value
Low-risk group			11.422	0.001	15.389	<0.0001
Intermediate-risk group	11.422	0.001			4.203	0.040
High-risk group	15.389	<0.0001	4.203	0.040		

**Table 5:** Prognostic value of the combination of the chemotherapy response system and normalization of CA125 for progression-free survival

	Unadjusted HR	95% CI	*P* value	Adjusted HR^a^	95% CI	*P* value
low-risk group	Reference			Reference		
intermediate-risk group	4.20	1.39 to 12.72	0.011	4.54	1.47 to 14.00	0.008
high-risk group	7.40	2.70 to 20.23	<0.0001	6.10	2.14 to 17.40	0.001

CI, confidence interval; HR, hazard ratio; ^a^ Adjusted HRs for progression-free survival were adjusted for R0 resection in interval debulking surgery (Yes vs No) and post-NACT hemoglobin levels (g/l).
