# Supplementary material for: The added value of CA125 normalization before interval debulking surgery to the chemotherapy response score for the prognostication of ovarian cancer patients receiving neoadjuvant chemotherapy for advanced disease
Source: J Cancer. 2021 Jan 1;12(3):946–53. doi: 10.7150/jca.52711 (PMC7778530; doi:10.7150/jca.52711)
Supplement: Supplementary file 1 — Supplementary tables. [file jcav12p0946s1.pdf]

Supplementary Table 1. CA125 normalization among patients achieving CRS3 (n=35)

	CA125 normalization	
	No	Yes
CRS3 without pCR, n (%)	15 (62.5)	9 (37.5)
CRS3 with CR, n (%)	3 (27.3)	8 (72.7)

CR, complete response; CRS, chemotherapy response score; pCR, pathological complete response;

Supplementary table 2. Comparison of recurrence-free survival using log-rank test with Bonferroni correction.

	CRS3+CA125≤35		CRS3+CA125>35		CRS1-2+CA125≤35		CRS1-2+CA125>35	
	Chi-Square	*P* value	Chi-Square	*P* value	Chi-Square	*P* value	Chi-Square	*P* value
CRS3+CA125≤35			10.634	0.001	11.706	0.001	16.918	<0.0001
CRS3+CA125>35	10.634	0.001			3.893	0.048	6.452	0.011
CRS1-2+CA125≤35	11.706	0.001	3.893	0.048			1.243	0.265
CRS1-2+CA125>35	16.918	<0.0001	6.452	0.011	1.243	0.265		

CRS, chemotherapy response score;
